# Malignancy risk stratification of thyroid nodules: comparisons of four ultrasound Thyroid Imaging Reporting and Data Systems in surgically resected nodules

**DOI:** 10.1038/s41598-017-11863-0

**Published:** 2017-09-14

**Authors:** Ying Wang, Kai-Rong Lei, Ya-Ping He, Xiao-Long Li, Wei-Wei Ren, Chong-Ke Zhao, Xiao-Wan Bo, Dan Wang, Cheng-Yu Sun, Hui-Xiong Xu

**Affiliations:** 1 Department of Medical Ultrasound, Shanghai Tenth People’s Hospital, Ultrasound Research and Education Institute, Tongji University School of Medicine, Shanghai, 200072 China; 2 Department of Medical Ultrasound, Yangpu Hospital, Tongji University School of Medicine, Shanghai, 200090 China; 3 Thyroid Institute, Tongji University School of Medicine, Shanghai, 200072 China; 4 Shanghai Center for Thyroid Diseases, Shanghai, 200072 China

## Abstract

To compare the efficiency of four different ultrasound (US) Thyroid Imaging Reporting and Data Systems (TI-RADS) in malignancy risk stratification in surgically resected thyroid nodules (TNs). The study included 547 benign TNs and 464 malignant TNs. US images of the TNs were retrospectively reviewed and categorized according to the TI-RADSs published by Horvath E *et al*. (TI-RADS H), Park *et al*. (TI-RADS P), Kwak *et al*. (TI-RADS K) and Russ *et al*. (TI-RADS R). The diagnostic performances for the four TI-RADSs were then compared. At multivariate analysis, among the suspicious US features, marked hypoechogenicity was the most significant independent predictor for malignancy (OR: 15.344, 95% CI: 5.313-44.313) (P < 0.05). Higher sensitivity was seen in TI-RADS H, TI-RADS K, TI-RADS R comparing with TI-RADS P (P < 0.05 for all), whereas the specificity, accuracy and area under the ROC curve (Az) of TI-RADS P were the highest (all P < 0.05). Higher specificity, accuracy and Az were seen in TI-RADS K compared with TI-RADS R (P = 0.003). With its higher sensitivity, TI-RADS K, a simple predictive model, is practical and convenient for the management of TNs in clinical practice. The study indicates that there is a good concordance between TI-RADS categories and histopathology.

## Introduction

Thyroid nodule occurs in about 20% to 76% of the adult population with wide use of imaging modalities and the incidence increases with age^1, 2^. Thyroid cancer is becoming increasingly prevalent in Eastern countries that the incidence of thyroid cancer has been rising 200% to 300% within the past 30 years^3^. Due to excellent spatial and temporal resolution, ultrasound (US) has become the first detection tool for the imaging examination of TNs, especially for the asymptomatic and nonpalpable TNs^4, 5^. The main clinical challenge in the treatment of these patients is to rule out malignancy. With the development of US techniques, including elastography^6, 7^ and contrast-enhanced US^8, 9^, diagnostic accuracy for thyroid nodule is increasing, however, conventional US is still the basic imaging modality since it is widely available and no special function is needed. For nodules with suspicious features on US, US-guided fine-needle aspiration cytology (FNAC) is always recommended to rule out malignancy, which is regarded as the most cost-effective modality for diagnosis of thyroid malignancy. In recent years, many versions^1, 2, 10–17^ of Thyroid Imaging Reporting and Data Systems (TI-RADSs) have applied US features to categorize TNs or recommend FNAC. By establishing a standardized language and coding system for radiologists and clinicians, TI-RADS not only stratifies the malignancy risk of the TNs, but also facilitates their clinical management and follow-up^10–13^.

Horvath *et al*.^10^ and Park *et al*.^11^ initially established TI-RADSs in 2009 with an intention to categorize different malignancy risks for TNs, which followed the concept of Breast Imaging Reporting and Data System (BI-RADS)^18^. The latter has been widely used as a standard method to describe mammographic and US features of breast lesions to correlate with breast malignancies. In 2011, Kwak *et al*.^12^ developed a risk stratification method for thyroid malignancy according to the number of suspicious US features including solid composition, hypoechogenicity, marked hypoechogenicity, microlobulated or irregular margins, microcalcifications, and taller than-wide shape. In the same year, Russ *et al*.^13^ established their TI-RADS classification and proposed an equation for predicting the probability of malignancy in TNs with and without elastography^19^. Nonetheless, the limitation of these studies^10–13^ is inherent due to using FNAC as the gold standard. FNAC diagnosis includes a percentage of undetermined lesions (the Bethesda category III, IV and V classifications) whose final results (benign or malignant) are questionable since surgery is not performed on all of them^20–22^. For the reason of sampling errors, cytological examination can not replace the pathological diagnosis. Due to its uncertainty, a validation study against a surgical reference standard to confirm the utility of previous four TI-RADS categories is mandatory in clinical practice. Therefore, we performed this retrospective study with surgical series of 1011 TNs with an aim to compare the efficiencies of the four TI-RADS classifcations in malignancy risk stratification of TNs, which would provide evidences to select an appropriate system under a special circumstance.

## Materials and Methods

This retrospective study was approved by our institutional review board and the requirement for informed consent from the patients was waived. The study was performed in accordance with relevant regulations.

### Patients

From September 2015 to December 2016, a consecutive of 1140 patients with TNs underwent thyroid US examinations and surgeries in this referral hospital. The exclusion criteria were as follows: (a) patients with incomplete US information (103 nodules); (b) nodules with undetermined pathological results (26 nodules). For analysis in patients with multiple nodules, we selected the nodules most suspicious for malignancy at US. When no nodules were suspicious for malignancy, the largest one would be evaluated. Finally, the study group consisted of 1011 pathologically proven nodules in 1011 patients (768 women and 243 men; mean age, 51.0  years ± 13.7; age range, 13–84 years). The diameter of the nodules ranged from 4.0 to 92.0 mm (mean, 18.4 mm ± 13.3).

### Conventional US

Conventional US was performed with Siemens S2000 (Siemens Medical Solutions, Mountain View, CA, USA; 5–14 MHz linear transducer), IU22 (Philips Medical Systems, Bothell, WA, USA; 5–12 MHz linear transducer) or Logiq E9 (GE Medical Systems, Milwaukee, WI, USA; 6–15 MHz linear transducer) instruments by three radiologists who were board-certified with more than 3 years of experience in thyroid US. All the US examinations were complied with the same protocol for thyroid scanning. The patient lied in the supine position, with their neck on a high pad. Conventional US images of the thyroid nodule were acquired by carefully scanning the thyroid and adjacent tissues both transversely and longitudinally. The US machine settings such as gain, focus, depth, time gain compensation, dynamic range, wall filter, color gain, were constantly adjusted until good quality US images were obtained. Conventional transverse, longitudinal and color Doppler US images were stored for each target nodule and then the images were recorded in the internal hard-disk for further off-line analysis. The nodule’s size was defined by the maximal diameter at US. The patients’ images with lymphadenopathy would also be stored.

### Image Interpretation

**Figure 1:**
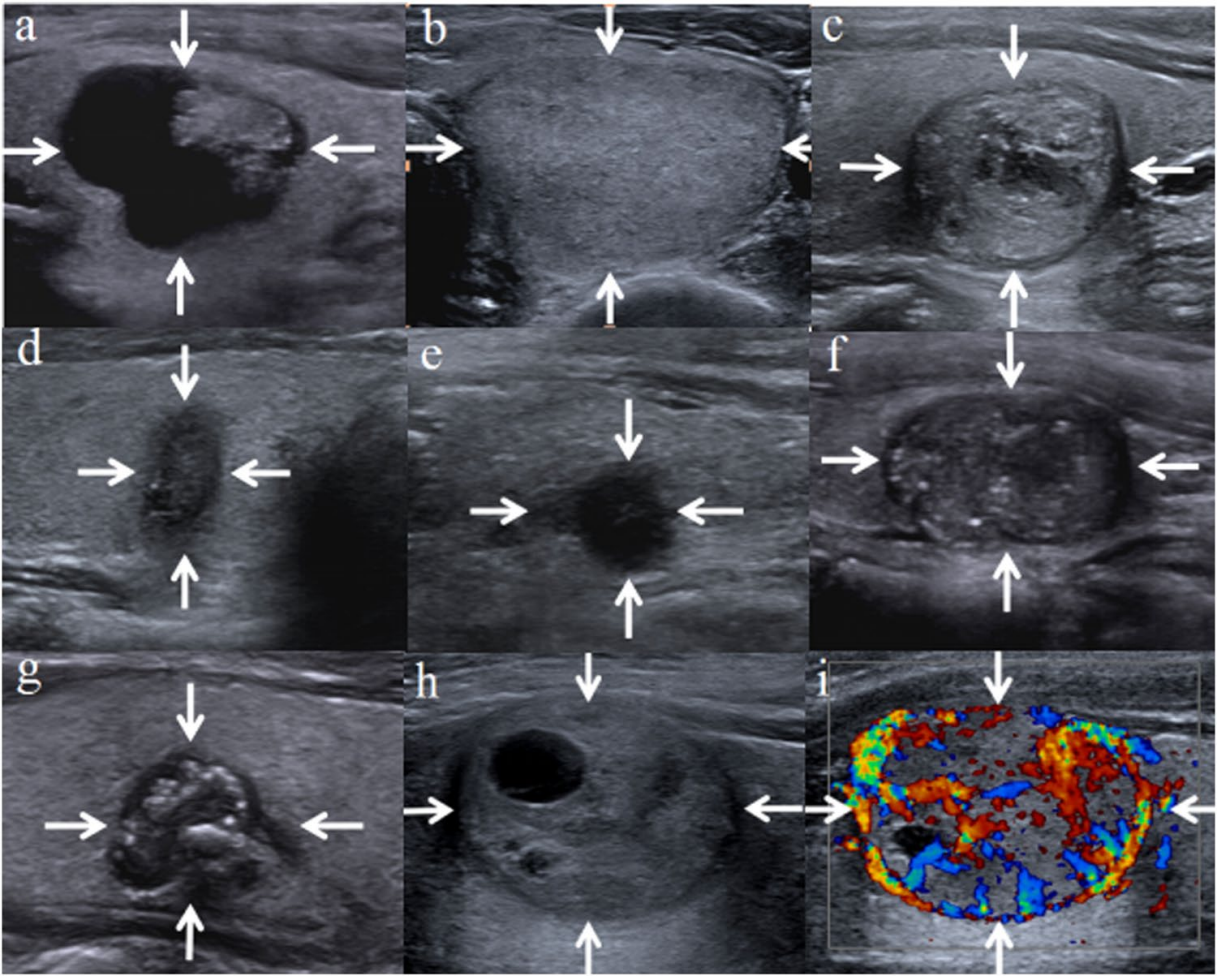
(**a**) Nodular goiter. Predominantly cystic nodule. TI-RADS H: 3; TI-RADS P: 1; TI-RADS K: 2; TI-RADS R: 3. (**b**) Follicular adenona. Solid and isoechoic nodule. TI-RADS H: 4a; TI-RADS P: 2; TI-RADS K: 4a; TI-RADS R: 3. (**c**) Papillary thyroid carcinoma. Solid and iso-hypoechoic nodule with microcalcification and hypoechoic halo, TI-RADS H: 4c; TI-RADS P: 4; TI-RADS K: 4b; TI-RADS R: 4b. (**d**) Papillary thyroid carcinoma. Solid and hypoechoic nodule with taller than wide shape, microlobulated margin, and microcalcification. TI-RADS H: 4c; TI-RADS P: 4; TI-RADS K: 5; TI-RADS R: 5. (**e**) Papillary thyroid carcinoma. Solid and marked hypoechoic nodule with microlobulated margin. TI-RADS H: 4b; TI-RADS P: 4; TI-RADS K: 4c; TI-RADS R: 4b. (**f**) Papillary thyroid carcinoma. Solid and hypoechoic nodule with disperse microcalcifications. TI-RADS H: 4c; TI-RADS P: 4; TI-RADS K: 4c; TI-RADS R: 4b. (**g**) Papillary thyroid carcinoma. Solid and hypoechoic nodule with microlobulated and mixed calcification. TI-RADS H: 4c; TI-RADS P: 5; TI-RADS K: 4c; TI-RADS R: 5. (**h**,**i**) Follicular thyroid carcinoma. Predominantly solid nodule with hypoechoic halo and hypervascular. TI-RADS H: 4c; TI-RADS P: 2; TI-RADS K: 3; TI-RADS R: 4a.

One of two radiologists who did not involved in image capture reviewed the US images and analyzed TI-RADS categories independently with 6 and 13 years of experience respectively in thyroid US. Patients’ medical information including previous imaging results and histopathological results were blinded to the two reviewers. They were firstly asked to read carefully the four TI-RADSs until they understood the TI-RADSs and then assessed the US characteristics defined by the authors. Then the two radiologists discussed a baseline consensus in lexicon for TI-RADS and US characteristics including location, composition, echogenicity, echostructure, margin, calcifcations, shape, vascularization, halo sign, capsule and cervical lymph node (Fig. 1). Location was categorized as right, left and isthmus. Composition was classified as solid (complete solid), predominantly solid (cystic portion ≤50%), predominantly cystic (cystic portion >50%)^11, 12^ and spongiform (aggregation of multiple microcystic components in more than 50% of the nodule) according to the ratio of the cystic portion to the solid portion in the nodule^10, 13^. Echogenicity was classified as hyper-, iso-, hypoechogenicity (compared with the normal thyroid gland) or marked hypoechoic (lower echogenicity than the adjacent strap muscle)^11–13^. Echostructure was categorized according to that the nodule echo was even or not. Heterogenous echoexture was defined as mixed echogenecity due to the aggregation of multiple microcystic components intervening the solid component^11^. Margin was classified as well circumscribed, microlobulated (presence of many small lobules on the surface of the nodule) or irregular margin and infiltrative (poorly defined margin with adjacent glanular structure)^11^. Calcifications were categorized as microcalcifications (≤1 mm in diameter, visualized with or without acoustic shadows), macrocalcifications (>1 mm in diameter, or rim calcification)^12^, mixed calcification (presence of microcalcifications and macrocalcifications at the same time)^23^, hyperechoic spot (present tiny bright reflectors with a clear-cut comet-tail artifact at conventional US)^10, 12, 13^, and no calcification. Kwak *et al*.^12^ regarded it as having microcalcification that a nodule had both types of calcifications, Park *et al*.^11^ defined microcalcifications as calcifications that were equal to or less than 0.5 mm in diameter. Shape was categorized as taller than wide (greater in its anteroposterior dimension than in its transverse dimension) or wider than tall^10–13^. Vascularization which was classified as avascular, hypovascularized (poorly blood flow signal), hypervascularized (highly vascularized on color Doppler) or penetrating vessels (vessels are not visualized in its interior, only afferent vessels that penetrate the lesion)^10^. Halo sign which was defined as a hypoechoic rim around a nodule included absent halo sign, partly halo and complete fine sign^11^. Capsule was defined as circinate hyperechogenicity around a nodule^10^. Cervical lymph node was classified as normal and lymphadenopathy including lymph nodes with minimal diameter > 6.0 mm or nodes with a absent hyperechoic hilum^10, 11^.

The TI-RADS categories were previously reported by Horvath E *et al*.^10^, Park *et al*.^11^, Kwak *et al*.^12^, Russ *et al*.^13^. We have summarized the classification of the different TI-RADS categories in Table 1.

**Table 1:** Four TI-RADS categories.

Scoring System and Category	Characteristics	Cancer risk	Recommendations
TI-RADS H^5,10*^			
1	Normal exam		
2	Hashimoto’s thyroiditis, typical De Quervain’s thyroiditis, Graves’s disease; Benign colloid lesions (Type 1 and 2 patterns); Intraparenchymal calcification without associated nodule; Aspirated nodule with benign result, concordant with its US image; Small hyperechoic pseudo-nodules in Hashimoto’s thyroiditis (“white knight”); Old colloid nodule in spontaneous regression (prior exam available, that shows the preexistence of a bigger colloid lesion on the same location); Situations, such as normal post-surgical control	Benign findings 0.0% malignancy	Follow-up
3	Typical hyperplastic colloid nodules with hyperechoic spots (Type 3 pattern); Hypoechoic pseudo-nodules in Hashimoto’s thyroiditis that for some reason (size, shape) appear to be different from the other thyroiditis focus dispersed within the parenchyma	Probably benign <5.0% malignancy	Follow-up/FNAC
4a	Solid or mixed hyper, iso, or hypoechoic nodule, with a thin capsule. Simple neoplastic pattern Hypoechoic lesion with infiltrative borders, without calcifications(de Quervain pattern) Hyper, iso, or hypoechoic, hypervascularized, encapsulated nodule with a thick capsule, containing calcifications (coarse or microcalcifications) (suspicious neoplastic pattern).	Low suspicion 5.0–10.0%malignancy	FNAC
4b	Hypoechoic, nonencapsulated nodule, with irregular shape and margins, penetrating. vessels, with or without calcifications (Malignant pattern A)	Intermediate suspicion 11.0–65.0% malignancy	FNAC
4c	The presence of micro and/or coarse calcifications and penetrating vessels increase suspicion (Malignant pattern A) Mixed or solid isoechoic nodule, non-encapsulated, vascularized with micro - or macrocalcifications (without hyperechoic spots, Malignant pattern C)	High suspicion 66.0–95.0% malignancy	FNAC
5	Nodules with malignant patterns (Types B and C); Adenopathies and ipsilateral suspicious nodules	Suggestive of malignancy > 95.0%	FNAC
6	FNAC-confirmed malignancy	100% malignancy	Surgery
**TI-RADS P** ^11*^			
0	Normal exam		
1	Cystic predominant, peripheral halo	Highly benign 0.0–7.0% malignancy	No additional US is recommanded if clinically not needed
2	Circumscribed margin, solid predominant, heterogeneous echotexture, iso- to hyperechogenecity, eggshell or macrocalcification	Probably benign 8.0–23.0% malignancy	Long-term US follow-up if clinicaly needed
3	Homogeneous echotexture, hypoechogenecity, circumscribed margin, solid, taller, without other US findings suggestive of malignacy	Indeterminate 24.0–50.0% malignancy	Aspiration and short-term (6 month) follow-up if nondiagnositic cytological result
4	One or two US findings suggestive of malignancy, such as markedly hypoechoic, microcalcification, not-circumscribed margin, and lymph node abnormality	Probably malignant 51.0–90.0% malignancy	Aspiration and immediate reaspiration if nondiagnostic FNAC result
5	More than three US findings suggestive of malignancy, such as markedly hypoechoic, microcalcification, not-circumscribed margin,and lymph node abnormality	Highly malignancy 1.0–100%	Consider surgery regardless of FNAC results
TI-RADS K^12*^			
1	Normal exam		
2	Predominantly cystic peripheral halo	Benign 0.0% malignancy	Follow-up
3	No suspicious US features	Probably benign 2.0–2.8% malignancy	Follow-up
4a	One suspicious US feature	Low suspicion for malignancy 3.6–12.7%	FNAC, ≥1.0 cm
4b	Two suspicious US features	Intermediate suspicion for malignancy 6.8–37.8%	FNAC ≥1.0 cm
4c	Three or four suspicious US features	Moderate concern but not classic for malignancy 21.0–91.9%	FNAC ≥1.0 cm
5	Five suspicious US features including solid, hypoechogenicity, microlobulated or irregular margins, microcalcifications, taller than-wide shape	Highly suggestive of malignancy 88.7–97.9%	FNAC ≥1.0 cm
TI-RADS R^13*^			
1	Normal exam		
2	Simple cyst Spongifrom nodule ‘white knight’ Isolated macrocalcification Nodular hyperplasia	Benign findings 0% malignancy	Follow-up
3	No sign of high suspicion: Regular shape and borders No microcalcifications and Isoechoic or Hyperechoic	Probably benign <2.0% malignancy	Follow-up
4a	No sign of high suspicion -Mildly hypoechoic	Mildly suspect 2.0–10.0% malignancy	FNAC
4b	One or two signs -No metastatic- lymph node	Highly suspect 10.0–95.0% malignancy	FNAC
5	Three to five signs ingcluding markly hypoechogenicity, microlobulated or irregular margins, microcalcifications, taller than-wide shape and/or -Metastatic -lymph node	Highly suspect >95.0% malignancy	FNAC

^*^Data are numbers of references.

### Statistical analysis

Statistical analyses were performed with SPSS software for Windows (version 20.0; Chicago, IL, USA) and MedCalc software (version 15.2, Mariakerke, Belgium). Independent two-sample t test was used to compare the continuous data including patient age and nodule size. Chi-square test was used to compare the categorical data including US features and patient sex. With adjustment for all variables, multivariate logistic regression analysis was performed to determine independent predictors for malignancy from the US characteristics that showed statistical significance. Odds ratios (ORs) with relative 95% confidence intervals (CIs) were also calculated to determine the relevance of all potential predictors for malignancy. The cut-off value for each TI-RADS category, was obtained from receiver operating characteristic (ROC) analysis when Youden index was maximum, as well as sensitivity and specificity. Positive predictive value (PPV), negative predictive value (NPV) and accuracy were all calculated by the diagnostic test 2 × 2 contingency tables. ROC curve analysis was performed to assess the diagnostic performance. The sensitivity and specificity were compared by Mcnemar test. Z test was applied to compare the area under the ROC curves (Azs). Statistical significance was determined at a P value less than 0.05.

Inter- and intra-observer agreement were assessed using the guideline of Landis and Koch for interpreting kappa values: slight agreement (0.00–0.20), fair agreement (0.21–0.40), moderate agreement (0.41–0.60), substantial agreement (0.61–0.80), and almost perfect agreement (0.80–1.00)^24^.

## Result

Of the 1011 TNs included in this study, 547 (54.1%) were diagnosed as benign and the remaining 464 (45.9%) were diagnosed as malignant. Mean age of the patients with nodules diagnosed as malignant was significantly younger than that of patients with nodules diagnosed as benign (46.5 years ± 14.1 [age range, 13–84 years] vs 54.3 years ± 12.3 [age range, 18–83 years], respectively; P < 0.001). Mean size of the TNs diagnosed as malignant was significantly smaller than that of nodules diagnosed as benign (11.7 mm ± 8.2 vs 24.0 mm ± 14.2, respectively; P < 0.001). Patient sex showed no significant difference between benign and malignant nodules, and the female-to-male ratioes were 3.18 (416/131) and 3.14 (352/112) respectively (P = 0.501). Location of the TNs was significantly different between benign and malignant masses, and isthmus is association with malignancy (P = 0.035) (Table 2). The 1011 TNs in 1011 patients were all diagnosed with histopathological examination after surgery, including conventional papillary thyroid carcinoma in 455 nodules, follicular thyroid carcinoma in seven nodules, medullary carcinoma in one nodule, and Hürthle cell carcinoma in one nodule, nodular goiter in 413 nodules, Hashimoto’s nodule in 51 nodules, follicular adenoma in 35 nodules, esinophilic cell adenoma in five nodules, adenomatous goiter in 43 nodules.

**Table 2:** Basic demographic characteristics and conventional US features in predicting thyroid malignancy.

Parameter	Benign n = 547)	Malignant (n = 464)	total	P Value
**Patient Characteristics**				
Gender				0.501
Male	131 (23.9)	112 (24.1)	243	
Female	416 (76.1)	352 (75.9)	768	
Age				<0.001
Mean(y)^#^	54.3 ± 12.3	46.5 ± 14.1		
Range(y)	18–83	13–84		
**Nodule**				
Size				<0.001
Mean(mm)^#^	24.0 ± 14.2	11.7 ± 8.2		
Range(mm)	4.0–92.0	4.0–61.0		
Location				0.035
Right	276 (50.5)	218 (47.0)	494	
Left	254 (46.4)	216 (46.6)	470	
Isthmus	17 (3.1)	30 (6.4)	47	
Composition				<0.001
Predominantly cystic	145 (26.5)	1 (0.2)	146	
Predominantly solid	97 (17.7)	11 (2.4)	108	
Solid	288 (52.7)	452 (97.4)	740	
Spongiform	17 (3.1)	0 (0.0)	17	
Echogenecity				<0.001
Iso-Hyperechogenicity	260 (47.5)	17 (3.7)	277	
Hypoechogenicity	279 (51.0)	390 (84.1)	669	
Marked hypoechogenicity	8 (1.5)	57 (12.2)	65	
Echostructure				<0.001
Homogeneous	100 (18.3)	135 (29.1)	235	
Heterogeneous	447 (81.7)	329 (70.9)	776	
Margin				<0.001
Well circumscribed	472 (86.3)	134 (28.9)	606	
Microlobulated or irregular	74 (13.5)	326 (70.2)	400	
infiltrative	1 (0.2)	4 (0.9)	5	
Calcification				<0.001
No calcification	408 (74.6)	190 (40.9)	598	
Macrocalcification	39 (7.1)	18 (3.9)	57	
Microcalcification	34 (6.2)	213 (45.9)	247	
Mixed calcification	7 (1.3)	43 (9.3)	50	
Hyperechoic spot	59 (10.8)	0 (0.0)	59	
Shape				<0.001
Wider than tall	522 (95.4)	317 (68.3)	839	
Taller than wide	25 (4.6)	147 (31.7)	172	
Vascularization				0.070
Avascular	215 (39.3)	200 (43.1)	415	
Hypovascular	223 (40.8)	200 (43.1)	423	
Hypervascular or penetrating vessel	109 (19.9)	64 (13.8)	173	
Halo sign				<0.001
Absent	414 (75.7)	420 (90.5)	834	
Partly	26 (4.8)	4 (0.9)	30	
Complete fine	107 (19.6)	40 (8.6)	147	
Capsule				<0.001
Absent	460 (84.1)	445 (95.9)	905	
Present	87 (15.9)	19 (4.1)	106	
Cervical lymph node				<0.001
Normal	537 (98.2)	410 (88.4)	947	
Lymphadenopathy	10 (1.8)	54 (11.6)	64	

Note. — Numbers in parentheses are percentages. ^#^Data are means ± standard deviations.

At univariate analysis, the following US features showed significant association with malignancy: solid composition, hypoechogenicity, marked hypoechogenicity, homogeneous echotexture, microlobulated or irregular margin, microcalcification, mixed calcifications and taller than-wide shape (all P < 0.05, Table 2). At multivariate analysis, among the suspicious US features, marked hypoechogenicity was the most significant predictor (OR: 15.344, 95% CI: 5.313–44.313), followed by mixed calcifications (OR: 13.753, 95% CI: 4.916–38.473), solid Composition (OR: 11.085, 95% CI: 1.393–88.218), hypoechogenicity (OR: 6.736, 95% CI: 3.416–13.282), microlobulated or irregular margin (OR: 4.951, 95% CI: 3.216–7.621), microcalcification (OR: 4.761, 95% CI: 2.772–8.178), taller than-wide shape (OR:2.630 95% CI: 1.489–4.647) (P < 0.05 for all, Table 3).

**Table 3:** Association between thyriod malignancy and various US features.

parameter	Univariate analysis			Multivariate analysis		
	β	OR (95% CI)	P Value	β	OR (95% CI)	P Value
Marked hypoechogenicity	4.691	108.971 (44.845–264.794)	<0.001	2.731	15.344 (5.313–44.313)	<0.001
Mixed calcification	2.580	13.191 (5.826–29.865)	<0.001	2.621	13.753 (4.916–38.473)	<0.001
Solid	5.427	227.569 (31.665–1635.510)	<0.001	2.406	11.085 (1.393–88.218)	0.023
Hypoechogenicity	3.062	21.379 (12.785–35.750)	<0.001	1.907	6.736 (3.416–13.282)	<0.001
Microlobulated or irregular	2.742	15.518 (11.302–21.306)	<0.001	1.600	4.951 (3.216–7.621)	<0.001
Isthmus	0.804	2.234 (1.201–4.157)	<0.001	1.592	4.911 (1.822–13.243)	0.002
Microcalcification	2.599	13.453 (9.010–20.085)	<0.001	1.561	4.761 (2.772–8.178)	<0.001
Taller than wide	2.270	9.683 (6.196–15.131)	<0.001	0.967	2.630 (1.489–4.647)	0.001

Note— β, regression coefficient; OR, odds ratio; CI, confidence interval.

The malignancy rates of four TI-RADSs were all with signifcant differences among categories (P < 0.001 for all). The TI-RADS categories whose malignancy rates are all at the range of the recommendtion except the categories of TI-RADS P 2, TI-RADS K 3, TI-RAD R 3 and TI-RADS R 4a. (Table 4). The correlation coeffcient of four TI-RADSs between category and malignancy rate was 0.712, 0.731, 0.775, 0.733 respectively.

**Table 4:** Comparison of malignancy rates with four TI-RADSs.

Scoring System and Category	Final Diagnosis^*^		Recommended Malignancy Risk (%)	Calculated Malignancy Rate (%)	P Value
	Benign (*n = *547)	Malignant (*n = *464)			
TI-RADS H					<0.001
2	67 (12.2)	0 (0.0)	0.0	0.0	
3	201 (36.7)	5 (1.1)	<5.0	2.4	
4a	121 (22.1)	11 (2.4)	5.0–10.0	8.3	
4b	125 (22.9)	177 (38.1)	11.0–65.0	58.6	
4c	30 (5.5)	188 (40.5)	66.0–95.0	86.2	
5	3 (0.6)	83 (17.9)	>95.0	96.5	
TI-RADS P					<0.001
1	198 (36.2)	2 (0.4)	0.0–7.0	1.0	
2	192 (35.1)	13 (2.8)	8.0–23.0	6.3	
3	81 (14.8)	62 (13.4)	24.0–50.0	43.4	
4	76 (13.9)	332 (71.5)	51.0–90.0	81.4	
5	0 (0.0)	55 (11.9)	91.0–100.0	100.0	
TI-RADS K					<0.001
2	154 (28.2)	0 (0.0)	0.0	0.0	
3	133 (24.3)	4 (0.9)	2.0–2.8	2.9	
4a	123 (22.5)	11 (2.4)	3.6–12.7	8.2	
4b	92 (16.8)	56 (12.1)	6.8–37.8	37.8	
4c	42 (7.7)	345 (74.3)	21.0–91.9	89.1	
5	3 (0.5)	48 (10.3)	88.7–97.9	94.1	
TI-RADS R					<0.001
2	68 (12.5)	0 (0.0)	0.0	0.0	
3	179 (32.7)	3 (0.6)	<2.0	2.6	
4a	214 (39.1)	42 (9.1)	2.0–10.0	16.4	
4b	80 (14.6)	299 (64.4)	10.0–95.0	78.9	
5	6 (1.1)	120 (25.9)	> 95.0	95.2	

^*^Data are numbers of patients, with percentages in parentheses.

**Figure 2:**
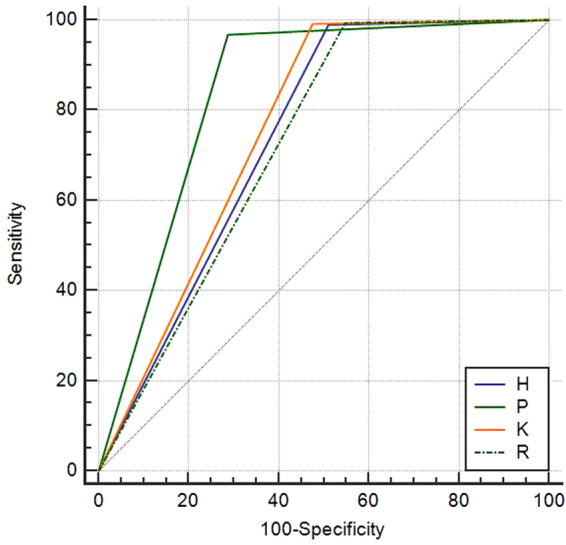
ROC curves of four TI-RADSs. Higher sensitivity was seen for TI-RADS H, TI-RADS K, TI-RADS R in comparison with TI-RADS P. Specifcity for the TI-RADS P was the highest compared with the other versions.

The categories were dichotomized into findings as positive and negative for FNA with the cut-off values and the diagnostic performances of four TI-RADSs were listed in Table 5. Higher sensitivity and negative predictive value were seen for TI-RADS H, TI-RADS K, TI-RADS R in comparison with TI-RADS P (P < 0.05 for all), whereas there were no significant statistical differences comparing with each orther (P > 0.05 for all). The specificity, accuracy and Az for TI-RADS P were the highest compared with the other systems (P < 0.05 for all). Higher specificity, accuracy and Az were seen for TI-RADS K compared with TI-RADS R (P = 0.003). The specificity, accuracy and Az of TI-RADS H and TI-RADS R were lower and no significant statistical difference was seen between them (P = 0.101). (Tables 5, 6, Fig. 2).

**Table 5:** Diagnostic performances of four TI-RADSs.

Parameter	TI-RADS H	TI-RADS P	TI-RADS K	TI-RADS R
Cut-off value	4a	3	4a	4a
Sensitivity (%)	98.9 (459/464)	96.8 (449/464)	99.1 (460/464)	99.4 (461/464)
Specificity (%)	49.0 (268/547)	71.3 (390/547)	52.5 (287/547)	45.2 (247/547)
PPV (%)	62.2 (459/738)	74.1 (449/606)	63.9 (460/720)	60.6 (461/761)
NPV (%)	98.2 (268/273)	96.3 (390/405)	98.6 (287/291)	98.8 (247/250)
Accuracy (%)	71.9 (727/1011)	83.0 (839/1011)	73.9 (747/1011)	70.0 (708/1011)
Az (95% CIs)	0.740 (0.711–0.766)	0.840 (0.816–0.862)	0.758 (0.730–0.784)	0.723 (0.694–0.750)

Note — Numbers in parentheses are raw data. Numbers in brackets are 95% confidence intervals. PPV = positive predictive value, NPV = negative predictive value. Az = area under ROC curve.

**Table 6:** Pairwise comparisons of four TI-RADSs.

	z statistic	P value		
		Az	Sensitivity	Specificity
H vs P	8.579	<0.001	0.021	<0.001
H vs K	2.158	0.031	1.000	0.042
H vs R	1.479	0.139	0.687	0.101
P vs K	8.556	<0.001	0.001	<0.001
P vs R	11.013	<0.001	0.002	<0.001
K vs R	2.957	0.003	1.000	0.003

Note— H = TI-RADS H; P = TI-RADS P; K = TI-RADS K; R = TI-RADS R.

Another 30 thyroid nodules were used for assessment of inter-observer agreement, and weighted kappa values of four TI-RADSs were 0.663 (95% CI: 0.446–0.830), 0.693(95% CI: 0.496–0.861), 0.748(95% CI: 0.565–0.914), 0.705 (95% CI: 0.492–0.873) respectively. Intra-observer agreement was assessed for one of two reviewers, and weighted kappa values of four TI-RADSs were 0.781 (95% CI: 0.581–0.951), 0.829(95% CI: 0.654–0.957), 0.874(95% CI: 0.727–1.000), 0.831 (95% CI: 0.651–0.958) respectively.

## Discussion

The TI-RADS H^10^ was a prospective study equation with 10 variables, defining categories 1, 2, 3, 4a, 4b, 5 and 6. Recently, they prospectively evaluated the diagnostic accuracy of their TI-RADS and modified category 4 to 4a, 4b, 4c^5^. They intergrated other factors including imaging findings, a nodule’s changes over time, previous FNAC results, different diffuse pathologies (e.g. Graves’ disease, Hashimoto’s thyroiditis, De Quervain thyroiditis) and varying clinical situations. These might be useful in management of different classifications of thyriod nodules. Calification (macrocalcification or microcalcification) and hypervascularity were significantly associated with malignancy in their study. In the present study, however, macrocalcification and hypervascular were not identified to be risk factors. The malignancy rate of each category is all at the range of the recommendtion.

Park *et al*. proposed their TI-RADS^11^ in a retrospective study with 12 aspects of TNs, adding size and lymph node abnormality and resulting in 5 categories: T-US 1–5 with an increasing the risk of malignancy. In the current study, size was also significantly different between benign and malignant nodules. Lymph node abnormality was a risk factor at univariate analysis whereas not at multivariate analysis. The result was probably attributed to interferences of other variables including microcalcification, microlobulated or irregular margin, or marked hypoechogenicity, which were all the malignancy risk factors. The malignancy risk was 6.3% among category 2 nodules which was lower than recommendtion (8.0 ~ 23.0%). US features mentioned in category 2 were all not risk factors in the present study, which was possibly the cause.

Kwak *et al*.^12^ created a predictive model based on US characteristics in a retrospective study that included 1658 nodules, considering that the risk of malignancy increased with the number of suspicious malignant US features including solid structure, marked hypoechogenicity, hypoechogenicity, microcalcification, microlobulated or irregular margin, and taller than wider shape. Our study was in concidence with them that solid composition was the predictor for carcinoma. During the process of reviewing images, we regarded the nodule as positive if there was a suspicious US features in it. It is practical and convenient for the management of TNs in clinical practice. The malignancy rate of each category were all at the range of the recommendtion.

Russ *et al*. published their TI-RADS system^13^ based on 24 US characteristics. Their study was based on a retrospective analysis of 500 FNAC nodules from one observer at a single institution. In 2013, they prospectively evaluated the diagnostic accuracy of their categories on 4550 nodules with and without elastography^19^. Other authors had adopted it and had developed their own classification systems^25, 26^. The malignancy risk was 2.6% (3/182) among category 3 nodules which was beyond the recommended malignancy rate (<2.0%). Surgical cases might be responsible for this result. The malignancy risk was 16.4% (42/256) among category 4a nodules in our study which was beyond the recommended malignancy rate (2.0~10.0%). This can translate to that hypoechogenicity, which is a US feature of 4a category, is malignancy risk factor at both univariate analysis and multivariate analysis. That the nodules in our study were surgical series might be one of the reasons.

The present study suggests that solid composition, hypoechogenicity, marked hypoechogenicity, homogeneous echotexure, microlobulated or irregular margin, microcalcification, mixed calcification and taller than-wide shape were independent US features in prediction of thyroid malignancy, consistently matching other published literatures^12, 14, 16, 27–29^. The current study had higher sensitivity and accuracy than those in previous studies^10–13^. The underlying reason is that our findings are specific to surgical patient cohorts with histopathology results, while the previous study focused on the TNs under the FNAC. TI-RADS P had higher diagnosis performance compared to the other three systems and had the higher specificity which is especially important in the management of TNs. Higher specificity can lower the rate of false-positive findings and eventually aviod overtreatment and reduce the number of unnecessary FNAC^25^. However, TI-RADS P had lower sensitivity relatively. As a tool used to select high-risk nodules for FNAC, higher sensitivity is very important in clinical practice. The malignancy nodules which were diagnosed benign category by Park *et al*. had the US features including hypoechogenicity with halo sign, macrocalcification or predominantly hyperechogenicity. Among these features, absent or present halo sign has no significant difference at multivariate analysis, hypoechogenicity is a important US feature in prediction of thyroid malignancy. These may be the reasons of its lower sensitivity. Although TI-RADS P stratified nodules into categories, it was not easy to assign every thyroid nodule into the equation proposed during reviewing the US images (e.g. predominantly solid nodule with halo sign). TI-RADS H, TI-RADS K and TI-RADS R achieved higher sensitivity to identify those nodules with high malignancy risk. TI-RADS K and TI-RADS R recommended FNAC for thyriod nodules with one or more suspicious US feature, which may have contributed to the higher sensitivity. Although Horvath E *et al*. intergrated many factors, this stereotypic US application was difficult for radiologists to use. Therefore, it was not easy to apply it to clinical practice^12^. The specificity of TI-RADS R was lower than that of TI-RADS K (P = 0.003). The specificity, accuracy and Az of TI-RADS H and TI-RADS R were lower and no significant statistical differences were found. Macrocalcification and iso-echogenicity are in malignant classification of TI-RADS H and TI-RADS R, respectively that may bring about their lower specificity. Comparing with the other three scoring systems, TI-RADS K was a simplicity and convenience predictive model based on five US characteristics, however, other three approaches had 10, 12, 24 aspects of TNs respectively^10–13^. As long as there is only one suspicious US feature in nodule, the nodule is positive with TI-RADS K. The TI-RADS categories whose malignancy rates are all at the range of the recommendtion except the categories of TI-RADS P 2, TI-RADS K 3, TI-RADS R 3 and TI-RADS R 4a. The results indicates that the TI-RADSs are appliable to both the general population with thyriod nodules and surgical series. The malignancy risks of TI-RADS K 3, TI-RADS R 3 and TI-RADS R 4a in surgical series are higher than in general population. The malignancy risk of TI-RADS P 2 in surgical series is lower than in general population. Inter-observer agreements were all substantial with four TI-RADSs. Perfect agreements of intra-observer agreements were obtained for TI-RADS P, TI-RADS K and TI-RADS R, whereas substantial agreement for TI-RADS H.

To our knowledge, this was the first study correlating US findings with ultimate histopathology in the surgical specimen to compare different TI-RADSs. Consequently, the study’s results of the diagnostic capacity of the classifications are not biased by the inherent inaccuracy of FNAC cytohistology results. FNAC diagnosis includes a percentage of undetermined lesions during general populations whose final results (benign or malignant) were unknown since surgery was not performed on all of them. Furthermore, in the surgical series, we collected information of the other nonsuspicious nodules present in surgical series, correlating pathology findings with nodules classified as benign patterns, that otherwise would confirm their absolute non-malignant aetiology.

Recently, with TI-RADS classifications being created, the TI-RADS system is continuously improved and modified according to new evidence, might including contrast-enhanced ultrasound^30, 31^, elastosonography findings^31, 32^, PET (positron emission tomography) findings, or other imaging techniques in the future. The TI-RADS system allows the clinicians to easily understand the malignancy risk of a thyroid nodule from the US report and make more correct treatment decisions such as follow-up, FNAC or operation.

Our research has several limitations. Firstly, the study was a surgical series that overrepresentation of cancers (45.9%) was present, compared to the FNAC-based series (i.e. 4.0–5.0%)^1^, which may lead to selection bias. However, at present, only histopathology is the gold standard for diagnosis of TNs^33^. Secondly, as a result of the retrospective research, various US machines and operators possibly limited the image interpretation by radiologists. However, all the US machines in this study were high-end instruments and were reviewed by experienced radiologists. In addition, the US images were scanned and stored under the same protocol, which reduced the influence to a minimal extent, still, a prospective study design is needed. Finally, it is a single center experience in a tertiary referral hospital and multi-center studies with large case series are mandatory. Further prospective studies are anticipated to verify our results.

## Conclusion

In conclusion, all the four TI-RADSs provide effective malignancy risk stratification for TNs. With its higher sensitivity, TI-RADS K, a simple predictive model based on five US characteristics, is practical and convenient for the management of TNs in clinical practice. The study also indicates that the TI-RADSs are appliable to surgical series, in addition to the general population.
